# Nanoparticles fabricated from the bioactive tilapia scale collagen for wound healing: Experimental approach

**DOI:** 10.1371/journal.pone.0282557

**Published:** 2023-10-20

**Authors:** Manal Shalaby, Dalia Hamouda, Shaimaa M. Khedr, Haitham M. Mostafa, Hesham Saeed, Ahmed Z. Ghareeb

**Affiliations:** 1 Medical Biotechnology Department, Institute of Genetic Engineering and Biotechnology, City of Scientific Research and Technological Applications, Alexandria, Egypt; 2 Centre of Excellence for Drug Preclinical Studies (CE-DPS), Pharmaceutical and Fermentation Industry Development Centre, City of Scientific Research and Technological Applications, New Borg El Arab, Alexandria, Egypt; 3 Department of Biotechnology, Institute of Graduate Studies and Research (IGSR), Alexandria University, Alexandria, Egypt; REVA University, INDIA

## Abstract

The creation of innovative wound-healing nanomaterials based on natural compounds emerges as a top research goal. This research aimed to create a gel containing collagen nanoparticles and evaluate its therapeutic potential for skin lesions. Collagen nanoparticles were produced from fish scales using desolvation techniques. Using SDS PAGE electrophoresis, Fourier transform infrared spectroscopy (FTIR) as well as the structure of the isolated collagen and its similarities to collagen type 1 were identified. The surface morphology of the isolated collagen and its reformulation into nanoparticles were examined using transmission and scanning electron microscopy. A Zeta sizer was used to examine the size, zeta potential, and distribution of the synthesized collagen nanoparticles. The cytotoxicity of the nanomaterials was investigated and an experimental model was used to evaluate the wound healing capability. The overall collagen output from Tilapia fish scales was 42%. Electrophoretic patterns revealed that the isolated collagen included a unique protein with chain bands of 126–132 kDa and an elevated beta band of 255 kDa. When compared to the isolated collagen, the collagen nanoparticles’ FTIR results revealed a significant drop in the amide II (42% decrease) and amide III (32% decrease) band intensities. According to SEM analysis, the generated collagen nanoparticles ranged in size from 100 to 350 nm, with an average diameter of 182 nm determined by the zeta sizer. The produced collagen nanoparticles were polydispersed in nature and had an equivalent average zeta potential of -17.7 mV. Cytotoxicity study showed that, when treating fibroblast cells with collagen nanoparticle concentrations, very mild morphological alterations were detected after human skin fibroblasts were treated with collagen nanoparticles 32 μg/ml for 24 hours, as higher concentrations of collagen nanoparticles caused cell detachment. Macroscopical and histological investigations proved that the fabricated fish scale collagen nanoparticles promoted the healing process in comparison to the saline group.

## Introduction

Given the complexity of chronic wounds environment, several multifunctional wound dressings have been proposed for the development of better tissue regeneration [1]. Advanced wound healing technologies involve the use of high-viscoelastic, biodegradable, and hydrophilic biomaterials that induce angiogenesis, decrease inflammation, regulate cell adhesion, and promote the growth of keratinocytes and fibroblasts [2,3]. As Collagen promotes the development of numerous cell layers in wounded skin, it has been proposed as an ideal candidate for wound dressing products [4]. Collagen-based dressings uniquely have the potential to reconstruct the intricate collagen architecture of original tissue ECM collagen and ECM-associated secondary components such as laminin, fibronectin, and glycosaminoglycans [5].

Another wound healing approach that would develop through technology is transforming materials that were designed as therapeutics to stimulate skin cell proliferation to be produced as nanoparticles utilizing various physicochemical techniques [6,7]. Furthermore, researchers have created nanoparticles of natural origin to address shortcomings and issues with current drug delivery methods, such as limited duration of action, need for repeated administration, erythema, burning sensation, and irritation [7]. A number of nano products have been created as permeable wound dressings; they are less cytotoxic than pure drugs and heal wounds from fungal infections more quickly with greater quality [8].

In this regard, collagen has been marketed as an attractive natural and safe substitute for drug delivery and therapeutic applications due to the existence of ionizable groups such as amino, phenol, guanidine, and imidazole [9,10]. Unfortunately, due to its large Mw of about 300 kDa, native collagen is unable to permeate the skin’s SC [10]. While collagen applications in wound healing have been broadly utilized, collagen nanomaterial reports are insufficient, as pure type-1 collagen from animal sources is expensive and has different forms.

For medical purposes, various forms of collagen were separated from marine sources, and the recovered protein was assessed using physicochemical characterization. Wound contraction and histological examinations were also utilized to test the efficacy of extracted collagen for wound healing. Assessments showed that collagen dressings enhanced wound resolution and closure. According to the findings, isolated fish scale collagen might be a promising alternative for wound healing [11].

Due to their high loading capacity, successful intracellular drug delivery, efficient cellular absorption, and sustained release in cytosols with efficient biodegradation in endosomes/lysozymes, nano-carriers were proposed as potential therapeutics [12,13].

Herein, we report a study on the preparation method and transformation of collagen into a new nanomaterial that influences cell propagation, movement, stratification, and ECM deposition, to enable the development of substitute skin tissues and wound dressings. The ARRIVE guidelines were used to conduct this investigation.

### Design and methodology

All experiments were carried out according to the relevant guidelines and ethical regulations of the Local Ethical Committee, City of Scientific Research & Applied Technology.

### Isolation of collagen from fish

Fish scale collagen (FSC) was extracted from tilapia fish scales obtained from sea capture thoroughly washed in flowing water as previously described [11]. The scales were then properly cleaned with distilled water, dried, and kept at -25°C until they were utilized.

The dried scales (5.0 g) were treated with 0.1 N NaOH for 3 days while changing the solution every day. Next, fish scales were cured with 0.5 M acetic acid for 3 days, and the extract was recovered by centrifugation at 50 000 g for an hour.

The salt of NaCl was added gradually to a final concentration of 0.9 M; the supernatants were pooled. To eliminate any salt, each pellet was rinsed and reprecipitated three times with pure water. The pellets were lyophilized after being suspended in 0.5 M acetic acid. Using a personal mill, the lyophilized samples were ground to a powder and sieved with a mesh (0.15 mm) sieve.

### Sodium Dodecyl Sulphate Gel Electrophoresis (SDS‑PAGE)

Collagen extracted from Tilapia was analyzed by performing a 10% SDS-PAGE according to Laemmli, 1970 [14]. For each well, 50 μg of protein was loaded. After electrophoresis, the gel was stained with Coomassie Brilliant Blue R-250 followed by de-staining in a solution of 10% (v/v) methanol and 10% (v/v) acetic acid.

### Spectrum analysis

Shimadzu FTIR 8400S, from Japan, was used to perform FTIR spectroscopy on a freeze-dried collagen sample. A sample of 10 mg was combined with 100 mg of dry potassium bromide (KBr) and compacted to form a disc with a diameter of 10 mm. The spectrum peak was scanned between 4500 and 500 cm^-1^ with a scanning rate of 45 scans per minute or 0.75 per second (0.75 Hz).

### SEM analysis

The microstructure of fish collagen and fish scale collagen nanoparticles were examined using SEM. The lyophilized collagen sample was punched and attached to an adhesive carbon stub. A tabletop SEM (JEOL 6340, Japan) was used for imaging at a voltage of 15 kV.

### Isolation and purification of collagen nanoparticles

The desolvation method was used for the preparation of collagen nanoparticles, that was first employed by Marty et al. [15]. This method employs the addition of absolute ethanol drop by drop to reach 50 ml as a desolvation factor to 50 ml of the collagen solution (0.5 g in 50 ml of 0.1 M acetic acid) the nanoparticle solution was then agitated for 3 hours at room temperature. This would alter the tertiary structure of collagen with the subsequent formation of collagen nanoparticles. Finally, an aqueous solution of glutaraldehyde (8%) was added and stirred for 24 hours at room temperature. The nanoparticles suspension was then purified by centrifugation (12000 ×*g*, 30 min) three times.

### Characterization of Nile fish scale‑based collagen nanoparticles

Light microscope, SEM, TEM, and DLS analyses were performed to characterize the formed Nile tilapia fish scale-based collagen nanoparticles. For better dispersion, the solution of Nile tilapia fish scale-based collagen nanoparticles was sonicated for 5 min to prepare the TEM sample.

The DLS technique was used to determine the size distribution and ζ potential of the produced Nile tilapia fish scale-based collagen nanoparticles. Measurements were carried out on a Malvern Zeta sizer (Malvern Instruments Corp., Malvern, United Kingdom) in solutions of pH = 5. All samples were diluted with Millipore-filtered (MF-Millipore™ Membrane Filters) deionized water to an appropriate scattering intensity.

### Collagen nanoparticles cellular treatment for Human Skin Fibroblast Cells (HSF)

Human fibroblast cells were obtained from the BJ-5ta (ATCC® CRL-4001TM) cell line and cultured in D-MEM media supplemented with 10% FBS in 25 cm^2^ flasks at 37°C and 5% CO_2_. Freeze-dried collagen nanoparticles were dissolved in D-MEM cell culture supplemented medium applied to cells at concentrations of 0.1, 1, 10, and 200 g/mL. Cells were washed twice with a sterile solution of phosphate-buffered saline (PBS) and collected for analysis at varied recovery periods (0, 24, 48, and 72 h). Complete media was used as a blank, whereas the negative controls in these studies were untreated cells.

### Preparation of collagen nanoparticles gel

Collagen nanoparticles 0.1 g was collected by centrifugation at 6000 rpm for 30 minutes with 1 ml of a platelet-rich plasma separation gel. The platelet-rich plasma separation gel is created from a resin that is made up of the following fundamental components in weight order: With a specific weight range of 1.068 to 1.080 at 25°C, 120 parts butyl acetate, 75–80 parts phenylethylene, 20–25 parts butyl acrylate, 5–12 parts acrylic acid, 2–2.5 parts azobisisobutyronitrile (AIBN), and 0.5–0.8 parts dodecyl mercaptan [16]. Thereafter, the collagen nanoparticle gel was collected for further testing.

### In vivo experimental study

The current research study was authorized by the board of the Institutional Animal Care and Use Ethics Committee of City of Scientific Research & Technology Applications (Application approval No. 53-3V-0122). The experimental research protocol was employed at the Pharmaceutical & Fermentation Industries Development Center and endorsed by the central committee. This study also followed the procedures established in the guide of the American Guide for Laboratory Animals Care & Use for the maintenance, management, sedation, analgesia, anesthesia, and euthanasia of laboratory animals.

Fifteen healthy indigenous rabbits (5–6 months old and weighing 1.5–2 kg) were used for the research. Animal groups were housed in isolated stainless-steel cages with regulated light and ambient temperature under a 12-hour light/dark cycle. Animals were clinically healthy and housed in the vivarium of the laboratory animal unit, Preclinical Studies Department, Pharmaceutical & Fermentation Industries Development Center (PFIDC), Borg El Arab, Alexandria.

All animals were numbered and weighed before being separated into three groups of five animals each: Group (I) acted as the vehicle control and received saline therapy, Group (II) received PRP gel treatment, and Group (III) received gel containing fish collagen nanoparticle treatment.

I/M injections of 2% Xylazine (1 mg/kg b.wt) and Ketamine HCl) 0.5 mg/kg b.wt (were used to sedate the rabbits. Each animal was separated, made to fast, and given a broad-spectrum antibiotic, Azithromycin 50 mg/kg, once before surgery and once per day in drinking water for three days afterward. To reduce pain stress on the animal, oral acetaminophen in 100 ml drinking water was given for 5 days after surgery.

The surgical region was shaved, and 4% alcohol-based iodine was applied as an antiseptic to the surgical site. Lidocaine 5% local analgesic sub-cutaneous injection at the incision site was applied. At the center of the shaved area on their upper backs, sterile surgical scissors were used to create a scar with a circular diameter of 2.5 cm and a full thickness open wound excision to the extent of subcutaneous tissue. For 21 days, all animal groups were maintained in their cages separately.

### Wound closure rates: Planimetry analysis

On postoperative days 0 to 21, a digital camera was used to take pictures of the experimental animals’ scars in comparison to a metric ruler. The assessment of calibrated wound closure in a two-dimensional plane and its measurement was defined by the appearance of wound edge closure and wound surface re-epithelialization. Four individual photo-micrographic measurements were taken of each rabbit.

### Standard histology of the wounded skin

Sodium pentobarbital was injected intravenously into the ear vein for the animals’ euthanasia before sampling. Sharp dissection was used to obtain a full-thickness skin flap tissue specimen from the operation site. Sections were then fixed using 10% formalin with subsequent paraffin processing. Serial sectioning was performed for paraffin sections harvested on Days 10 and 21, and H &E staining was performed for representative sections.

### Statistical analysis

IBM SPSS Statistics for Windows, version 25 (IBM Corp., Armonk, NY, USA) was used to analyze all data (α = 0.05). Quantitative data were expressed as mean and standard deviation. One way ANOVA test was used for comparing the three studied groups and followed by a Post Hoc test (Tukey) for pairwise comparison. The significance of the obtained results was judged at the 5% level.

## Results

**Fig 1:**
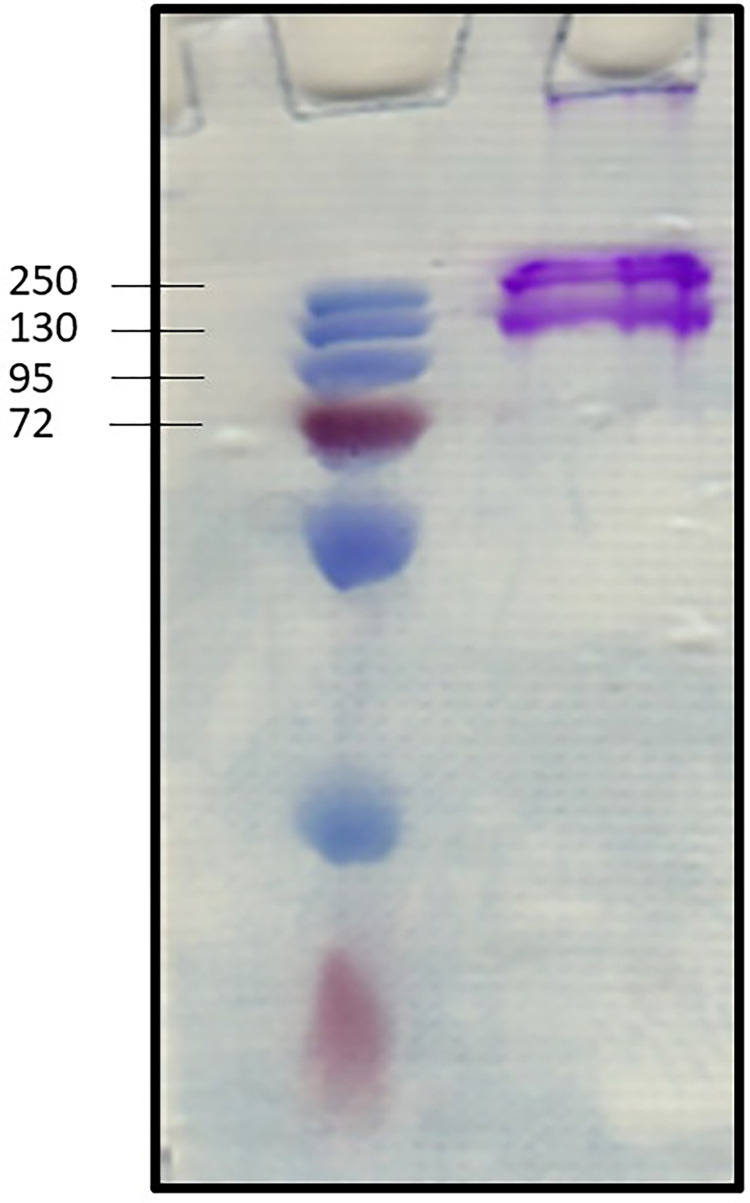
SDS (10%) of fish collagen isolated from tilapia (Lane 2). Lane 1 represents the pre-stained protein molecular weight marker.

The produced matter contained 91.2% of protein. Lyophilized collagen of 42 grams was produced from 100 g of fish scale. Sodium dodecyl sulfate-polyacrylamide gel electrophoresis was used to assess collagen source material purity and breakdown. The electrophoretic patterns of tilapia show characteristic protein bands corresponding to an α chain in the range of 126–132 kDa, respectively; the beta band was situated at a higher value (approximately 255 kDa) (Fig 1). A characteristic electrophoretic profile is comparable with other fish species [17].

### FTIR

**Fig 2:**
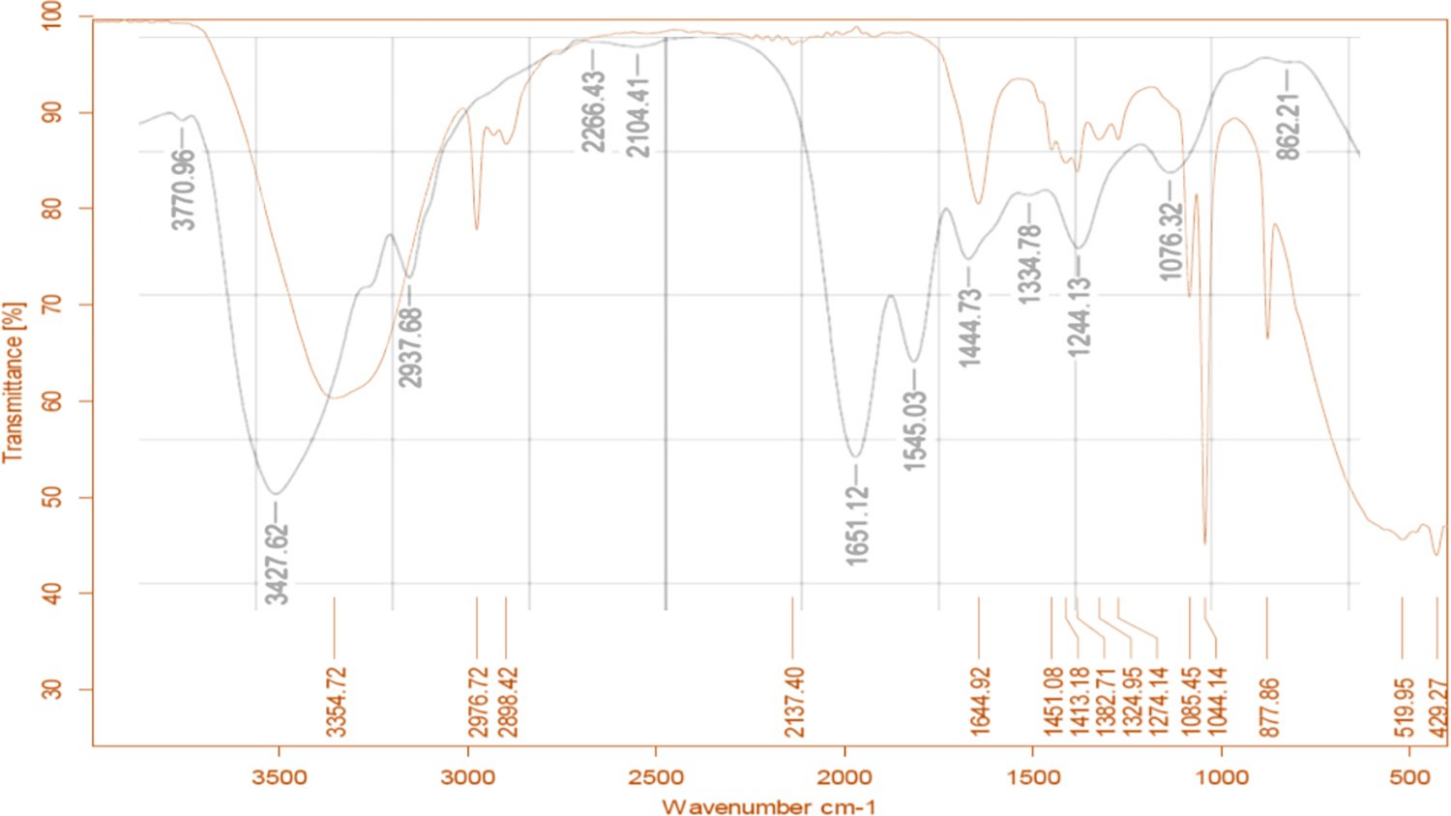
FT-IR spectrum of extracted fish scale collagen and collagen nanoparticles. Extracted collagen is represented by a black line and collagen nanoparticles are represented by a red line.

The secondary structure of collagen found in fish scales and the synthesized nanoparticles was examined using FTIR (Fig 2). In FTIR spectra between 450 and 4000 cm^-1^, the primary absorption bands of amides A (3427 cm^-1^), B (2937 cm^-1^), I (1651 cm^-1^), II (1545 cm^-1^), and III (1244 cm^-1^) could be detected [17]. The spectra of collagen NPs exhibited a slight shift in the characteristic bands of collagen as shown in Fig 2. In addition, some bands showed intensity differences (Fig 2), since it was possible to note a strong decrease in intensity in the amide II (42% decrease) and amide III (32% decrease) bands.

### Collagen fibers modulation for nanoparticles formation

**Fig 3:**
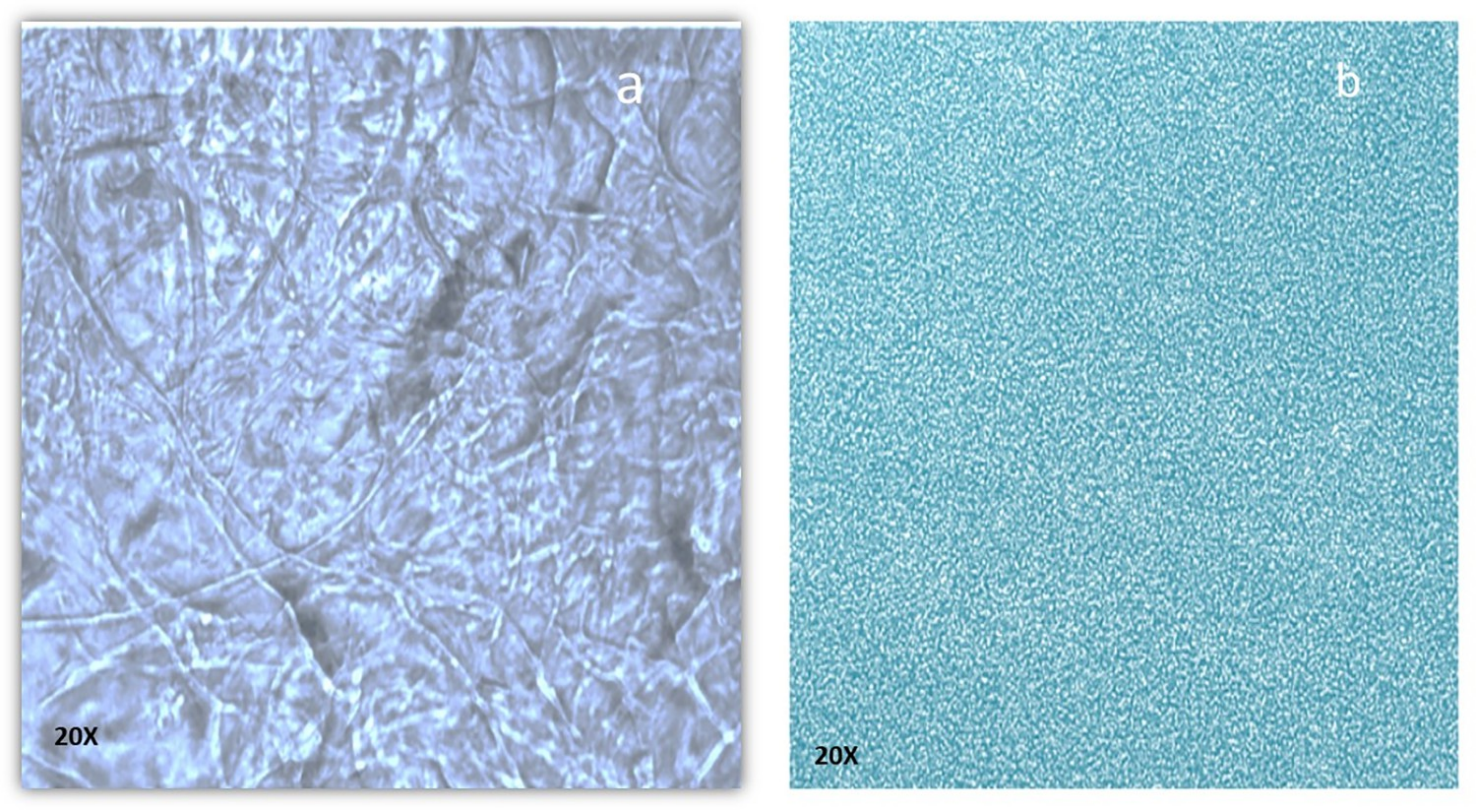
Light Microscopy Images of Collagen Fibers Prepared from the Scales of Tilapia (a) Collagen nanoparticles prepared using desolvation-based method (b).

The purified collagen was examined with optical microscopy to determine the presence of fibrils collagen structure (Fig 3A), which was curbed to tiny spheres upon desolvation (Fig 3B). The creation of nanoparticles could be indicated by the collagen solution having a milky appearance after the slow addition of ethanol. The effect of temperature and pH on nanoparticle size has been investigated; showing that the optimum temperature was 37°C while the optimum pH was 5.

### Scanning electron microscopy (SEM) analysis of nanoparticles

**Fig 4:**
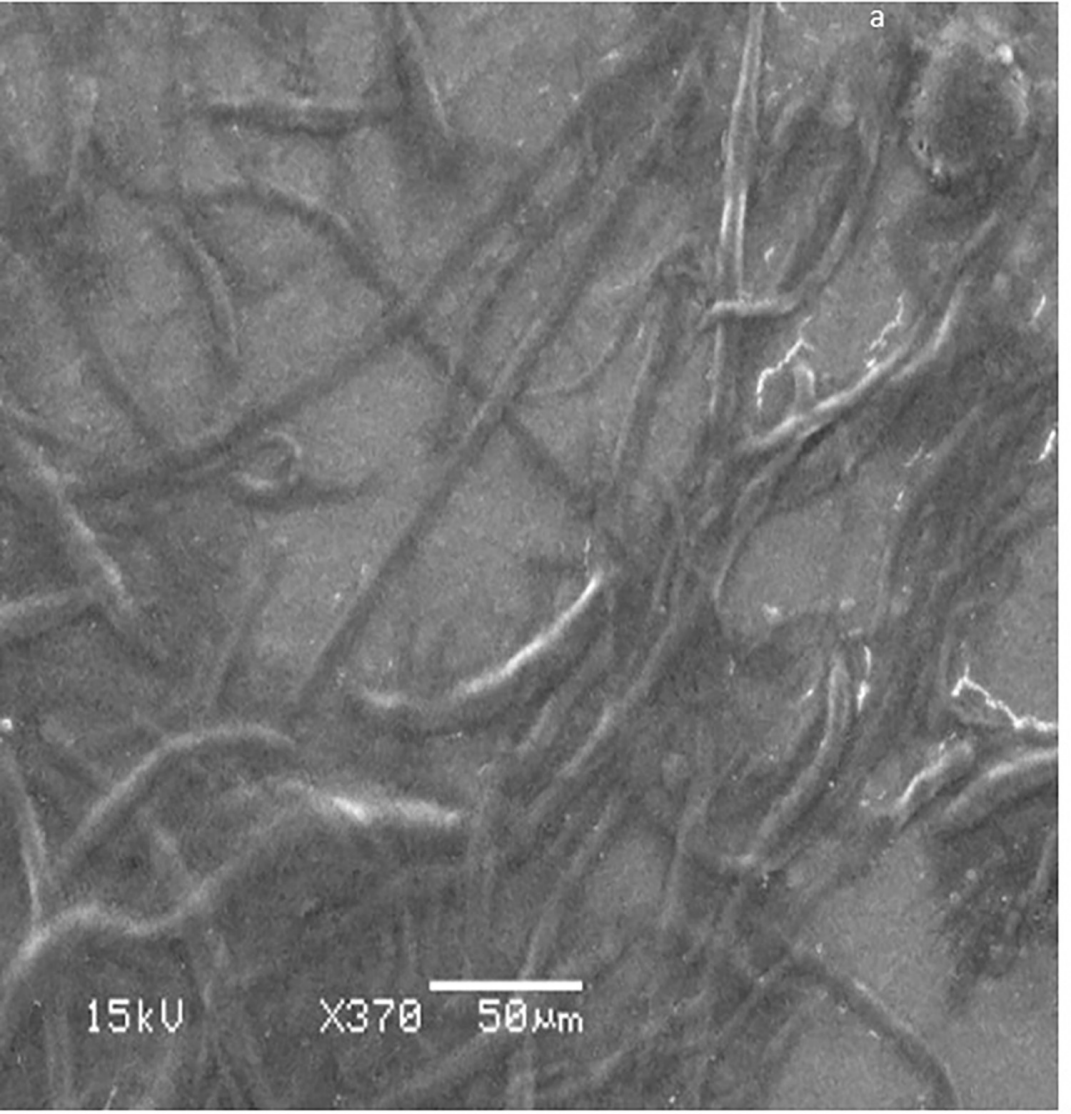
SEM observation of fish scale collagen (X370). The fibrillar structure of collagen is illustrated.

**Fig 5:**
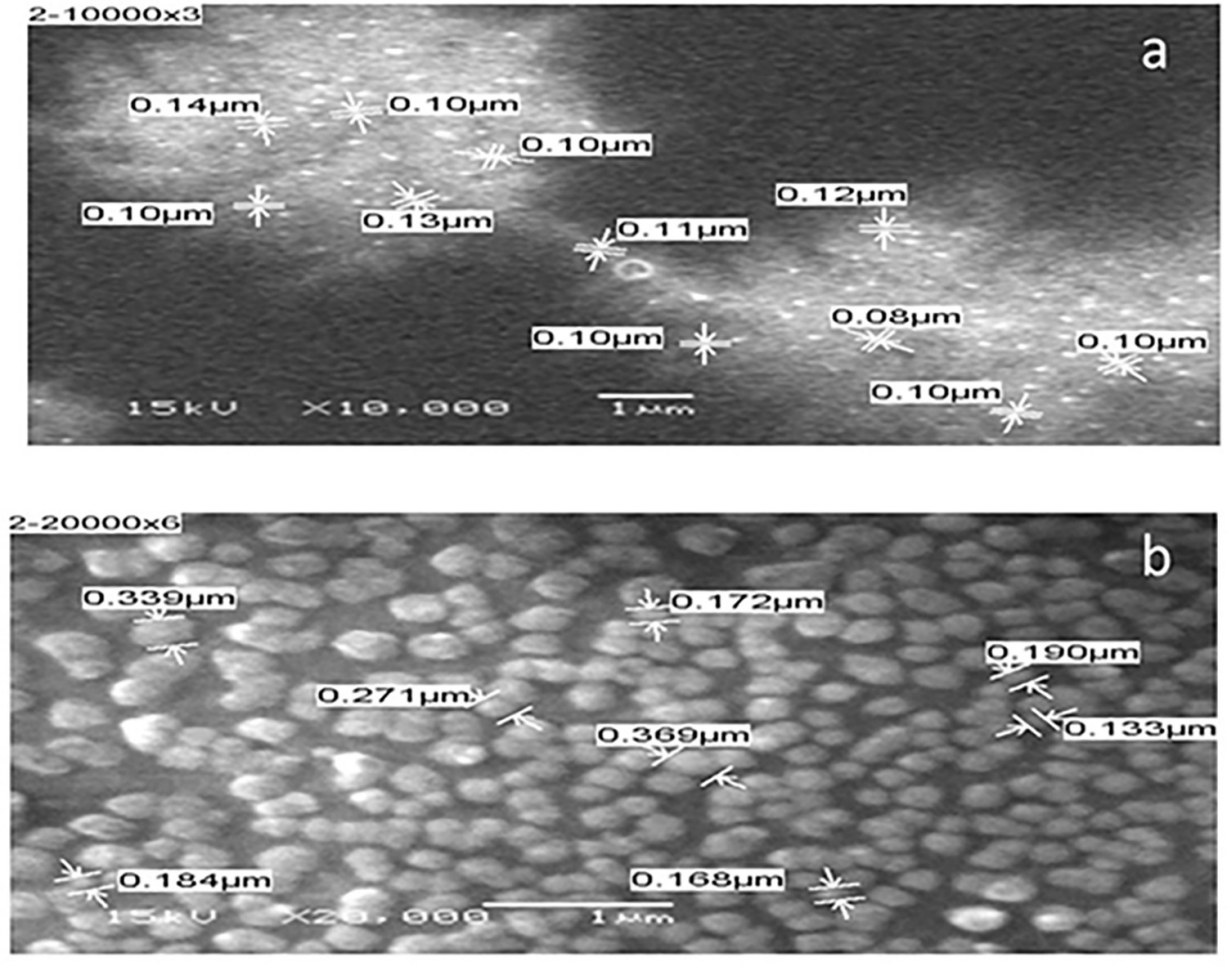
SEM observation of collagen nanoparticles. Collagen nanoparticle size at different magnifications;10,000 X (a) and 20,000 X (b).

SEM microscopy 370X revealed the fibrillar structure of collagen (Fig 4). The size of the articulated nanoparticle is between 100 and 350 nm at a magnification of 20000X (Fig 5).

### Transmission Electron Microscopy (TEM)

**Fig 6:**
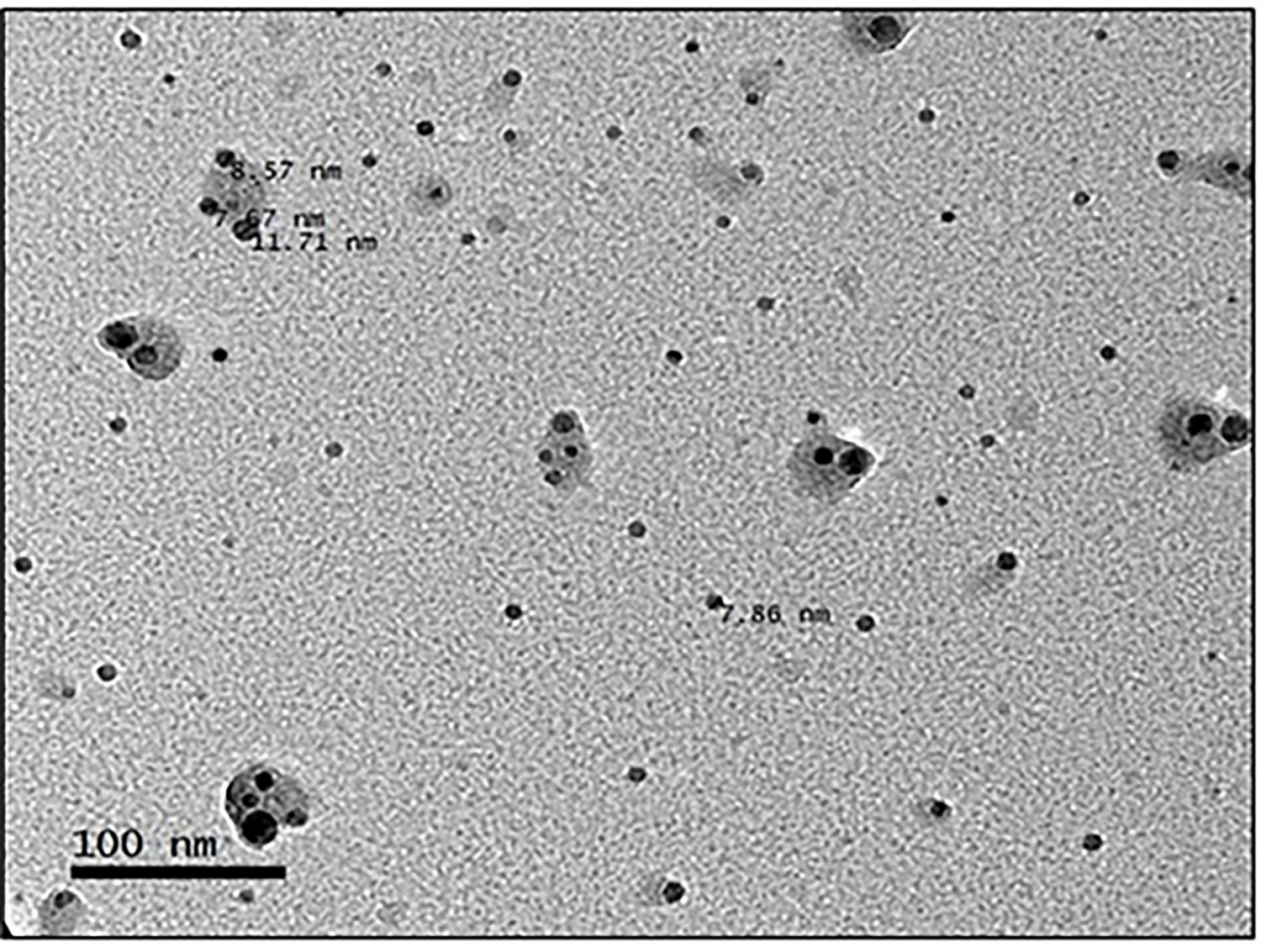
TEM observation of collagen nanoparticles. Collagen nanoparticles’ size, shape, and distribution are illustrated.

TEM images of collagen nanoparticles are presented in Fig 6, showing that nanoparticles are spherical in structure, having a size of 7.67, 8.57, and 11.71 nm. The surface of most of the nanoparticles is decorated with what seems like a protein shell, forming a biologically active protein corona.

### Determination of collagen nanoparticles’ size, potential, and distribution

**Fig 7:**
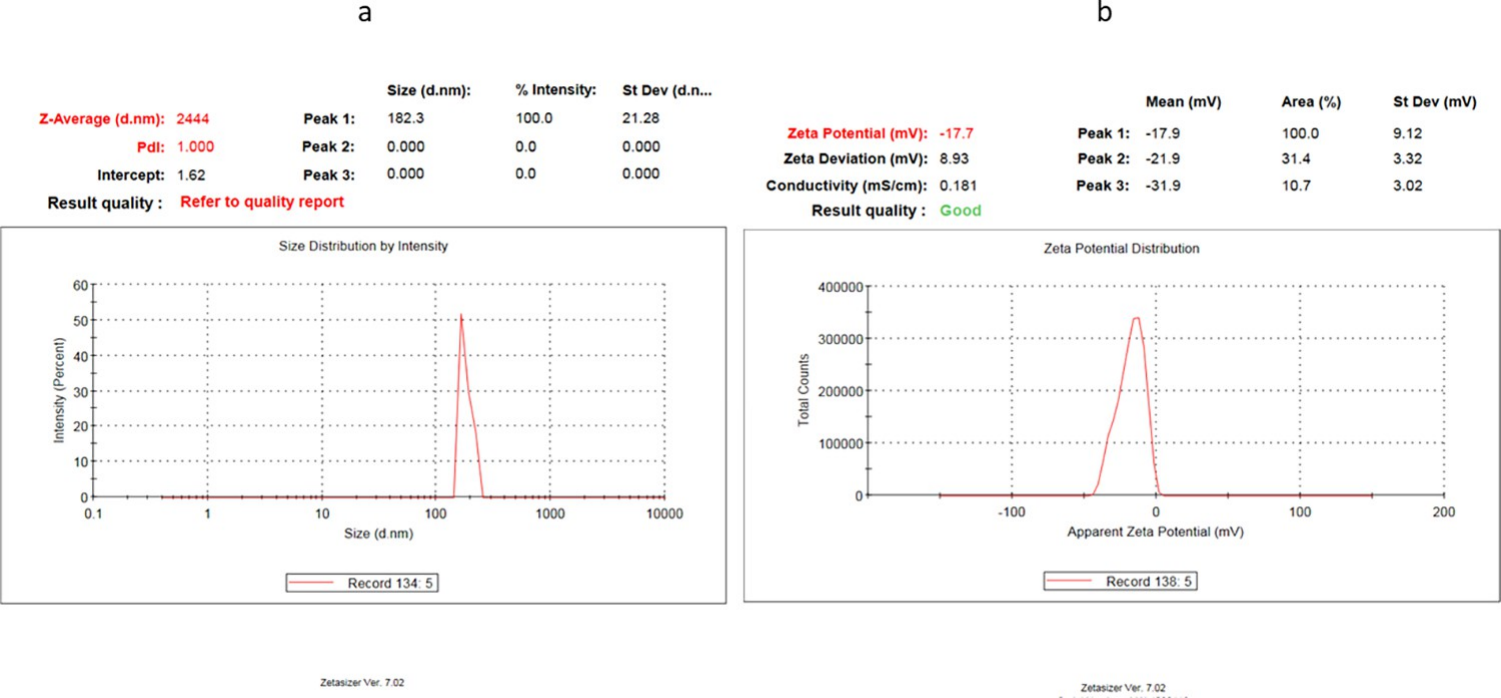
DLS analysis of collagen nanoparticles. Collagen nanoparticle sizes are represented as peaks (a) and collagen particle potential (b).

Collagen nanoparticles obtained were polydispersed in nature, with an average diameter of 182 nm and a comparable average zeta potential value of -17.7 mV (Fig 7). The polydispersity index (PDI) of the synthesized nanoparticles was 1, indicating a broad size distribution of the synthesized collagen nanoparticles.

### Human skin fibroblast containing collagen nanoparticles

**Fig 8:**
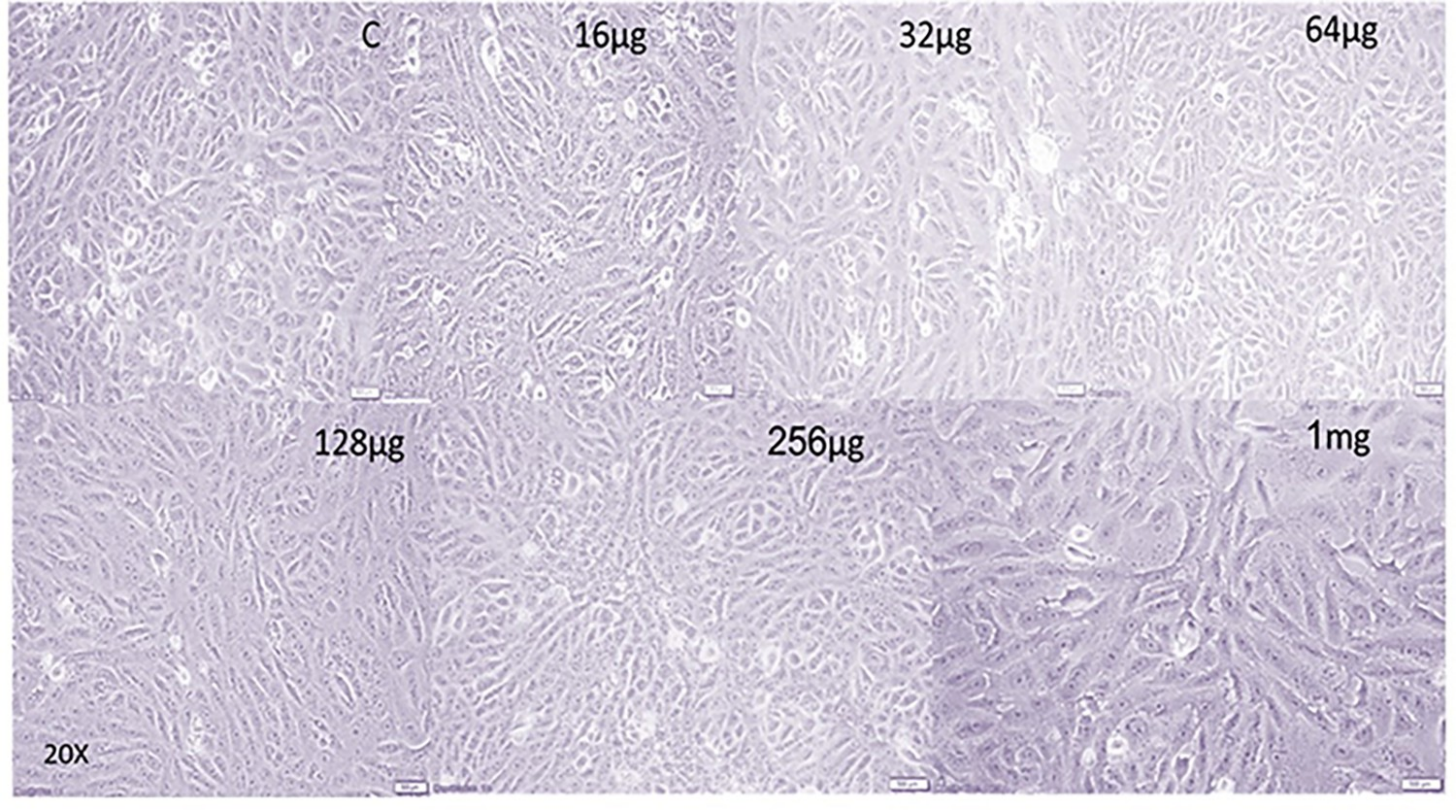
Light microscope image (10X) of Human Skin Fibroblast Cells (HSF). The cell was subjected to different concentrations (16 μg, 32 μg, 64 μg, 128 μg, 254 μg and 1 mg) of collagen nanoparticles.

HSF in its natural state is composed of polygonal, adhering cells that form a confluent monolayer (Fig 8C). Our results showed that treating fibroblast cells with collagen nanoparticle concentrations less than 64 μg/ml for 24 hours resulted in no or very mild morphological alterations, indicating that the cells were alive (Fig 8). Treatment of fibroblasts with larger quantities of collagen nanoparticles (64 μg/ml), on the other hand, dramatically increases the refractivity of the cell nucleus. Treatments with concentrations of 64 μg/ml and higher demonstrated morphological changes accompanied by increased cell-to-cell contact, implying that the cells started to separate from the base. There were also more cells with visible multinuclear, abnormal vacuoles, or particles in cellular plasma, as well as an increase in elongated cell numbers and visible filopodia.

### Testing the wound healing potential of collagen nanoparticles

#### *In vivo* wound healing assessment

**Fig 9:**
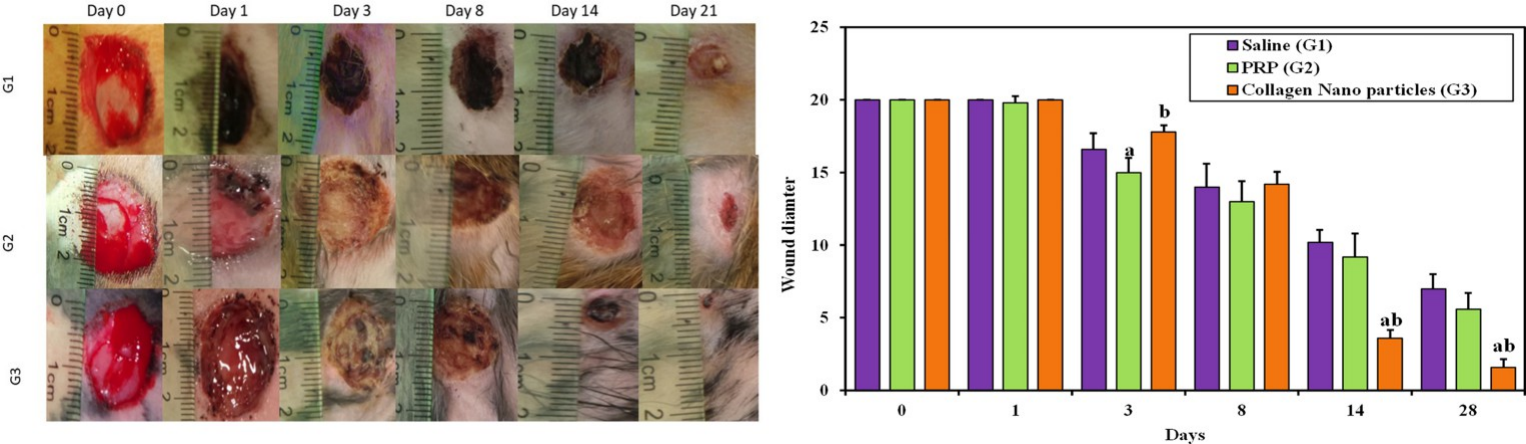
**a. Digital Photos Showing Macroscopic Wound Size Subjected to Different Treatments.** As compare Wounds subjected to different treatments compared with an initial value at Day 0 where (G1) saline control group & (G2) represents the group treated with PRP gel and (G3) is the group treated with gel mixed with collagen nanoparticles. **b**. **Chart Showing the Normalized Average Wound Size for all Study Groups.** a: Significant with **Control.** b: Significant with **PRP.** Wound size was measured in all groups over the 20 days study period.

In comparison to saline, PRP gel (G2) and gel mixed with collagen nanoparticles (G3) demonstrated effective wound sealing within 7–14 days of damage, for quicker and better re-epithelization. It also improved wound closure and had no contamination complications during the healing process (Fig 9A).

The macroscopic examination of the wounded area (Fig 9A) aimed to detect probable erythema near the lesion, the presence of extreme exudate, and the preservation of wound humidity. In comparison to saline wound healing, collagen nanoparticles restored the injured tissues. A collagen nanoparticle-based dressing was demonstrated to be suitable for dry wound cleaning and facilitated autolytic debridement. This dressing was permeable, did not react with the injured tissue, left little residue on the wound, and aided in wound re-epithelialization. On the third day, all wounds had shrunk in comparison to the operation day; nevertheless, variations could be seen when compared to the saline group gel (G2). In collagen nanoparticles groups (G3), the wound began to heal up with new tissue, and new skin formed over this tissue. The margins of the incision in collagen nanoparticles groups (G3) drew inward as it healed, and the wound shrank, displaying a crust all around the wound with granulated tissue detected, suggesting that it had dried.

All important elements of the wound assessment process; exudation, inflammation, and microbial contamination; were remarkably decreased upon treatment with collagen gel. Wound diameter was calculated and compared for all groups as shown in Fig 9B. On Day 8, a thin tissue layer formed over the whole surface of the wounds, mostly those treated with collagen gel. At this point, all the lesions had shrunk. The lesions’ size had shrunk by Day 14, and full re-epithelization had been seen in the collagen-based group. When compared to the gel (G3) and the control saline groups, the collagen nanoparticles promoted quicker wound healing and repairing. Collagen gel nanoparticles demonstrated improved wound closure due to their ease of application and high adherence to the wound bed. Statistical analysis of the wound size in all the tested groups revealed that at the third day of injury PRP gel significantly improved wound healing as compared to the control group (p-value < 0.001), rats treated with collagen gel nanoparticles has better wound healing as compared to PRP gel group (p-value < 0.001). Collagen nanoparticles gel group significantly improved wound healing as compared to control and PRP gel groups at the 14^th^ and 28^th^ day of injury (p-value < 0.001) (Fig 9B).

### Histopathological analysis

During the healing process, inflammation is the body’s self-defense mechanism for removing essential stimuli such as irritants, pathogens, or damaged cells. Many cells, including macrophages, eosinophils, and neutrophils, participate in the pathogenesis of chronic inflammation by the generation of inflammatory cytokines.

**Fig 10:**
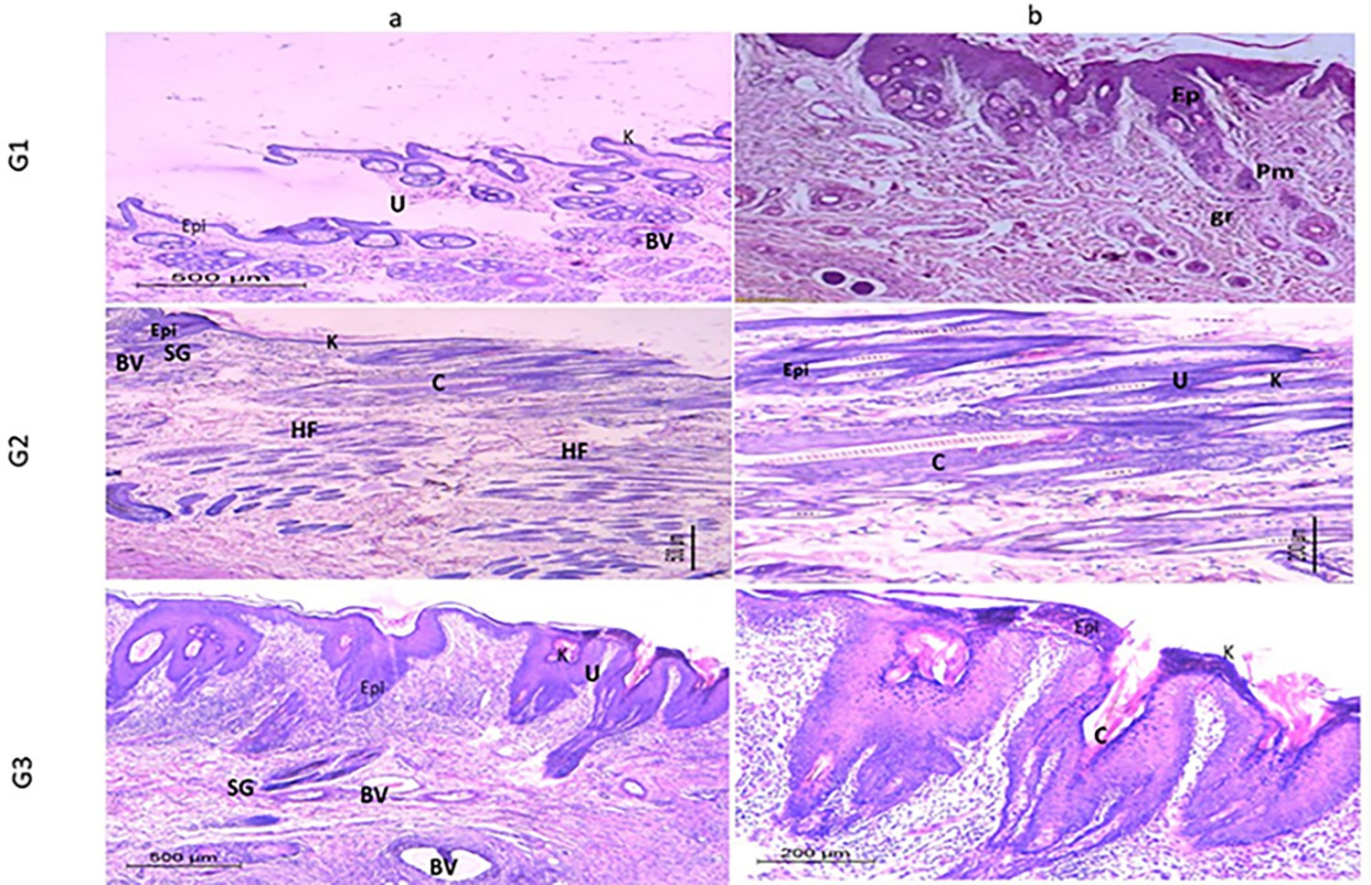
Histological analysis of wound treatments (10X). Wounds stained with H&E staining treated with PRP gel (GII), gel supplemented with collagen nanoparticles (GIII), and saline as negative control (Saline) (GI) on the 10th day. Epi: Epidermis; K: Keratin layer; BV: Blood vessels; HF: Hair follicles; SG: Sebaceous glands; U: Ulcer; C: Collagen; Connective tissue (CT) a and b are the images with 10x and 20x magnification.

**Fig 11:**
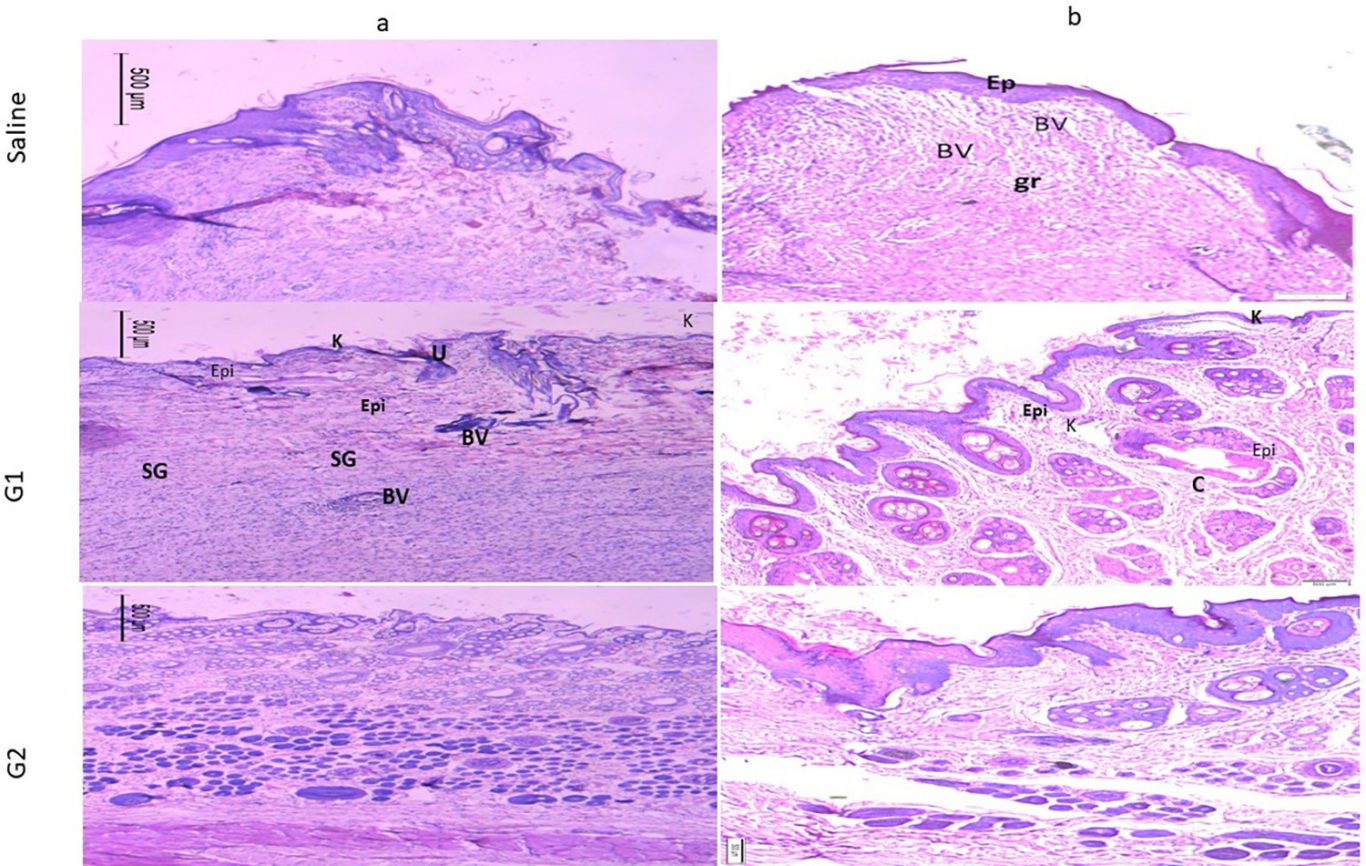
Histological analysis of wound treatments (20X). Wounds stained with H&E staining treated with PRP gel (GII), gel supplemented with collagen nanoparticles (GIII), and saline as negative control (Saline) (GI) on the 21st day. Epi: Epidermis; K: Keratin layer; BV: Blood vessels; HF: Hair follicles; SG: Sebaceous glands; U: Ulcer; C: Collagen; Connective tissue (CT) a and b are the images with 10x and 20x magnification.

Figs 10 and 11 show the image of the histopathological examination of the wounded areas of skin from different animals of the treatment groups on Day 10 and Day 21 respectively, of H &E stained (X10 & X20). Wounded skins demonstrated a varying response to the treatment, at which the finest wound healing efficacy and full epithelization were verified for the collagen nanoparticle gel G III group followed by the PRP gel GII group. On the 10^th^ and 21^st^ days, the wound region in the treated groups exhibited no ulcer with virtually normal skin covered in dense connective tissues, and a fresh dermal layer.

The image of the histological analysis of the wounds from different treatment groups on Day 10 and Day 21 is shown in Figs 10 and 11. Animals from the untreated wounds Group GI (Saline group) had ulceration with significant infiltration of inflammatory cells or granulation tissue, but no newly developed blood vessels or hair follicles in H&E-stained sections. The dermis was disorganized, with the generation of an inflammatory cap and the presence of a gap, which indicates the incomplete healing of the induced ulcer.

As with typically healed skin, the tissues have a well-formed epidermis (external epithelium made up of 2–3 cell layers) and dermis (connective tissue layer). The most fundamental elements of the rabbit’s skin, such as hair follicles (HF), sebaceous glands (SG), and blood vessels (BV), were also visible in the skin sections. Angiogenesis has been shown to have a critical role in the regeneration of both the epidermis (EPI) and dermis (D) containing hair follicles. The promising action of collagen nanoparticle gel could be attributed to quicker healing and epithelization.

## Discussion

In the current study, we created a medicinal nano-biomaterial from waste collagen and assessed its effectiveness in healing rabbit wounds. Here, we applied the desolvation approach, and as demonstrated by SEM, FTIR and DLS analysis, the identifiable collagen fibers transformed to accumulations that were spherical in shape (182 nm). FTIR analysis revealed the related shift in the amide I, amide II and amide III bands with a noticeable decrease in intensity in the amide II (42% decrease) and amide III (32% decrease) bands indicating changes in the C–N and/or NH bonds, due to interactions of different groups on collagen and confirming the formation of collagen nanoparticles. Changing the amount of ethanol or the rate at which it is added to the procedure changes the solubility of proteins, affecting the size of the NPs generated. Langer et al discovered that using the desolvation method for the conformational changes of the molecule to produce NPs is primarily due to the manual and dropwise addition of ethanol [18]. Collagen nanoparticle synthesis depends on controlled precipitation, in which the force of precipitation, caused by the removal of water molecules, is balanced by intermolecular charge repulsions caused by the changing pH. Aside from that, an increase in collagen water-holding ability at low pH may result in an increase in the ethanol concentrations necessary.

These findings are closely related to the research by Jahanban-Esfahlan et al, who used the desolvation process to produce albumin nanoparticles with a controlled particle size of roughly 100 nm for drug delivery applications [19]. Combining collagen with nanoparticle features (such as better surface-area-to-volume ratio, high porosity, improved mechanical capabilities, and good ability to distribute bioactive compounds) may speed up wound healing and increase skin regeneration [20].

The bulk of the nanoparticles also had a protein shell on their surface, creating a protein corona that is physiologically active, according to TEM analyses. Depending on the situation, collagen nanoparticle biomedical applications may profit from or suffer from protein corona’s biological impacts [21]. The thin layer of protein corona seems to lessen nanoparticle adhesion and aggregation, inhibiting the identification of macrophages or, in the worst situation, the formation of a thrombus [22].

In order to assess the collagen nanoparticle’s surface charge and determine its physical stability, one important characterisation approach is to measure its zeta potential. Negative or positive nanoparticles with high zeta potential are thought to be electrically stable systems, whereas particles with low zeta potentials tend to agglomerate or coagulate, which results in poor physical stability. The strong negative potential value of collagen nanoparticles (-17.7 mV), as indicated by DLS, enhances their long-term stability, superior colloidal nature, and high dispersion due to negative–negative repulsion [23]. According to some reports, nanoparticles’ negative potential induces electrostatic repulsions between individual particles, which prevent aggregation. This level of stability is necessary to prevent thrombosis and the aggregation of nanoparticles [24].

The created collagen nanoparticles are typically smaller than 200 nm and larger than 5 nm, which makes them a potential drug delivery method for entering cells via endocytosis, reduced-size particles have a larger surface area and a greater propensity to penetrate the skin’s barrier layers [25,26]. Due to electrostatic interactions, they might conveniently attach to the cell membrane as cationic nanoparticles [27].

In our research, it was discovered that collagen nanoparticle-infused gel had beneficial impacts on the healing of incision wounds. Our results are in agreement with those of Mondal et al., who developed gold-loaded hydroxyapatite collagen nano-biomaterials with improved properties that encouraged cellular adhesion, growth, and proliferation while having bioactive and biocompatibility properties [27].

*In vivo* testing demonstrated that collagen nanoparticles improved wound healing and produced substantial changes in tissue repair therapies on the tenth day and twenty-first day after damage. Increased cellular proliferation and collagen production at the wound site demonstrated the extraordinary wound-healing ability of collagen nanoparticles. The collagen improvement of wound contraction may have increased the number of myofibroblasts or enhanced their contractile abilities, even though myofibroblasts are regarded to be crucial for the centripetal movement of the wound margin [28].

The basic objective of a wound dressing, to keep the wound clean and free of external pollutants, appears to be achieved by these nanoparticles [29]. It also keeps the site hydrated, promoting healing, and preventing the wound’s origin from being exposed. In addition to being able to transfer active compounds to aid in the healing process, the new nanoparticles have the capacity to protect the wound environment [29]. Compared to the control group, collagen nanoparticles increased wound healing, indicating that they may be employed in wound dressing for skin regeneration.

## Conclusion

The present research highlights novel approaches for wound healing, where collagen nanoparticles, derived from fish scales, were demonstrated to have qualities similar to type I collagen. The synthesized nanoparticles were studied for cell cytotoxicity in the presence of human skin fibroblast cell lines, and no cytotoxic effects were identified for collagen nanoparticles up to 64 μg/ml loading. A little toxicity was observed when the collagen nanoparticles loading reached 1mg/ml. According to macroscopical and histological analyses, these nanoparticles had connected effectively with the wound region and quickly penetrated the epidermal layer at the wound site because of their nano size and high surface area-to-volume ratio. The synthesized and well-characterized collagen nanoparticle system could be cheap and useful for drug delivery as well as a suitable extracellular matrix for tissue engineering and biomedical applications.

## Supporting information

S1 File Supplementary file 1 Wound size and their row data analysis in the different studied groups. (DOCX)
