# Supplementary material for: Nanoparticles fabricated from the bioactive tilapia scale collagen for wound healing: Experimental approach
Source: PLoS One. 2023 Oct 20;18(10):e0282557. doi: 10.1371/journal.pone.0282557 (PMC10588885; doi:10.1371/journal.pone.0282557)
Supplement: S1 File — (DOCX) [file pone.0282557.s001.docx]

| Control group | | | | | | | |
| --- | --- | --- | --- | --- | --- | --- | --- |
| days | R1 | R2 | R3 | R4 | R5 | Mean | SD |
| 0 | 20 | 20 | 20 | 20 | 20 | 20 | 0 |
| 1 | 20 | 20 | 20 | 20 | 20 | 20 | 0 |
| 3 | 17 | 18 | 16 | 17 | 15 | 16.6 | 1.140175425 |
| 8 | 14 | 15 | 13 | 16 | 12 | 14 | 1.58113883 |
| 14 | 10 | 11 | 10 | 11 | 9 | 10.2 | 0.836660027 |
| 21 | 7 | 8 | 6 | 8 | 6 | 7 | 1 |

**Supplementary file Wound size and their row data analysis in the different studied groups**

| PRP gel | | | | | | | |
| --- | --- | --- | --- | --- | --- | --- | --- |
| days | R1 | R2 | R3 | R4 | R5 | mean | SD |
| 0 | 20 | 20 | 20 | 20 | 20 | 20 | 0 |
| 1 | 20 | 20 | 19 | 20 | 20 | 20 | 0.447213595 |
| 3 | 16 | 14 | 15 | 16 | 14 | 15 | 1 |
| 8 | 14 | 12 | 11 | 14 | 14 | 14 | 1.414213562 |
| 14 | 10 | 8 | 7 | 11 | 10 | 10 | 1.643167673 |
| 21 | 6 | 5 | 4 | 7 | 6 | 6 | 1.140175425 |

| Collagen Nano | | | | | | | |
| --- | --- | --- | --- | --- | --- | --- | --- |
| days | R1 | R2 | R3 | R4 | R5 | mean | SD |
| 0 | 20 | 20 | 20 | 20 | 20 | 20 | 0 |
| 1 | 20 | 20 | 20 | 20 | 20 | 20 | 0 |
| 3 | 18 | 17 | 18 | 18 | 18 | 18 | 0.447213595 |
| 8 | 15 | 13 | 14 | 15 | 14 | 14 | 0.836660027 |
| 14 | 4 | 3 | 4 | 4 | 3 | 4 | 0.547722558 |
| 21 | 2 | 1 | 1 | 2 | 2 | 2 | 0.547722558 |

**Comparison between the three studied groups according to wound**

| **Wound** | **Control** | **PRP** | **Colagen nano** | **F** | **p** |
| --- | --- | --- | --- | --- | --- |
| **0** | 20 ± 0 | 20 ± 0 | 20 ± 0 | – | – |
| **1** | 20 ± 0 | 19.8 ± 0.45 | 20 ± 0 | 1.0 | 0.397 |
| **3** | 16.6 ± 1.1 | 15^a^ ± 1 | 17.8^b^ ± 0.45 | 11.840^*^ | 0.001^*^ |
| **8** | 14 ± 1.6 | 13 ± 1.4 | 14.2 ± 0.84 | 1.192 | 0.337 |
| **14** | 10.2 ± 0.84 | 9.2 ± 1.6 | 3.6^ab^ ± 0.55 | 51.297^*^ | <0.001^*^ |
| **28** | 7 ± 1 | 5.6 ± 1.1 | 1.6^ab^ ± 0.55 | 45.308^*^ | <0.001^*^ |

Data was expressed using Mean ± SD. 5 replica for each group

**F**: **F for One way ANOVA test**, Pairwise comparison bet. each 2 groups was done using **Post Hoc Test (Tukey)**

p: p value for comparing between the studied groups

*: Statistically significant at p ≤ 0.05

a: Significant with **Control**

b: Significant with **PRP**
